# Supplementary material for: An ECG-based artificial intelligence model for assessment of sudden cardiac death risk
Source: Commun Med (Lond). 2024 Feb 27;4:17. doi: 10.1038/s43856-024-00451-9 (PMC10899257; doi:10.1038/s43856-024-00451-9)
Supplement: Supplementary file 2 — Supplementary Information [file 43856_2024_451_MOESM2_ESM.pdf]

# Supplementary Information

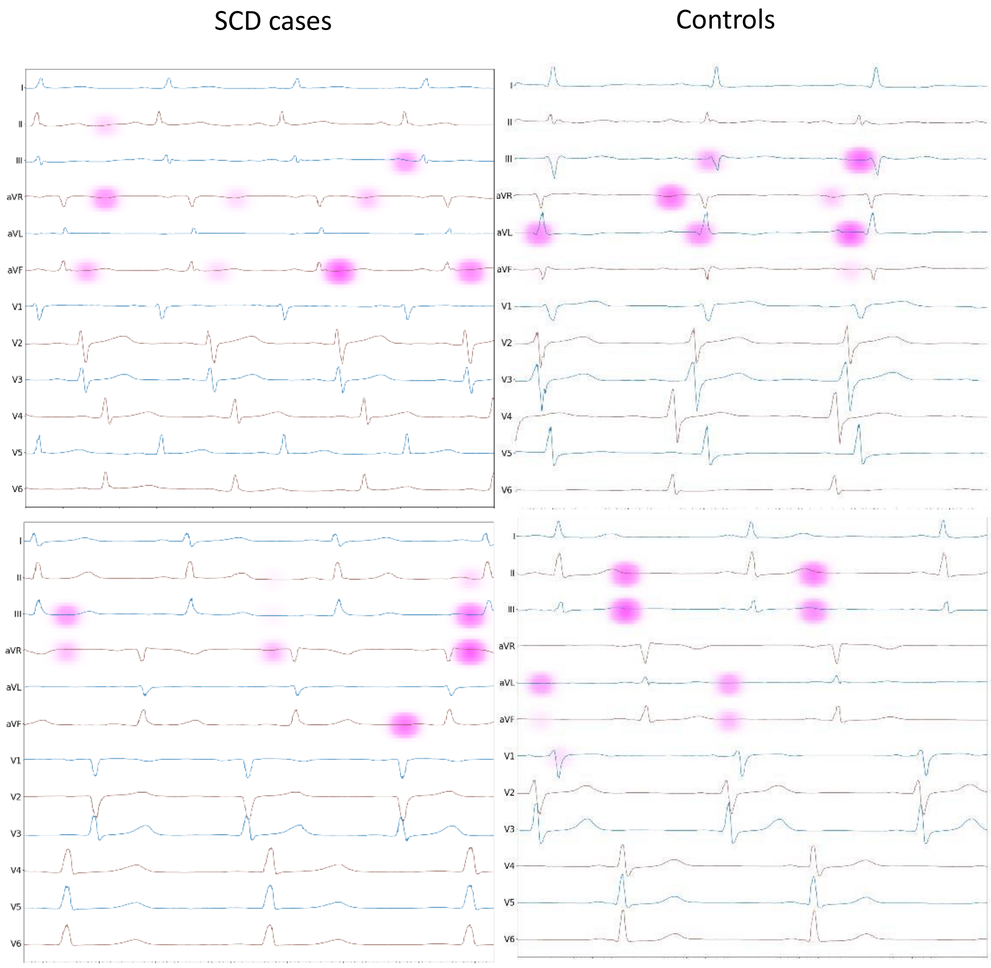


**Supplementary Figure**. The Linear Interpretable Model-Agnostic Explanations (LIME) map of 12-lead ECG for two SCD cases and two controls in the external cohort. LIME highlights features that were important for the model decision.
