# Supplementary material for: An ECG-based artificial intelligence model for assessment of sudden cardiac death risk
Source: Commun Med (Lond). 2024 Feb 27;4:17. doi: 10.1038/s43856-024-00451-9 (PMC10899257; doi:10.1038/s43856-024-00451-9)
Supplement: Supplementary file 3 — Reporting Summary [file 43856_2024_451_MOESM3_ESM.pdf]

nature portfolio

Sumeet S. Chugh MD
Cedars-Sinai Medical Center
Division of Artificial Intelligence in Medicine
127 S. San Vicente Blvd.
AHSP Pavilion Suite A3100
Los Angeles, CA 90048
Corresponding author(s): sumeet.chugh@cshs.org

Last updated by author(s): Dec 20, 2023

Reporting Summary

Nature Portfolio wishes to improve the reproducibility of the work that we publish. This form provides structure for consistency and transparency in reporting. For further information on Nature Portfolio policies, see our Editorial Policies and the Editorial Policy Checklist.

Statistics

For all statistical analyses, confirm that the following items are present in the figure legend, table legend, main text, or Methods section.

n/a	Confirmed	
☐	☒	The exact sample size (*n*) for each experimental group/condition, given as a discrete number and unit of measurement
☐	☒	A statement on whether measurements were taken from distinct samples or whether the same sample was measured repeatedly
☐	☒	The statistical test(s) used AND whether they are one- or two-sided<br>*Only common tests should be described solely by name; describe more complex techniques in the Methods section.*
☐	☒	A description of all covariates tested
☒	☐	A description of any assumptions or corrections, such as tests of normality and adjustment for multiple comparisons
☐	☒	A full description of the statistical parameters including central tendency (e.g. means) or other basic estimates (e.g. regression coefficient) AND variation (e.g. standard deviation) or associated estimates of uncertainty (e.g. confidence intervals)
☒	☐	For null hypothesis testing, the test statistic (e.g. *F*, *t*, *r*) with confidence intervals, effect sizes, degrees of freedom and *P* value noted<br>*Give P values as exact values whenever suitable.*
☒	☐	For Bayesian analysis, information on the choice of priors and Markov chain Monte Carlo settings
☒	☐	For hierarchical and complex designs, identification of the appropriate level for tests and full reporting of outcomes
☒	☐	Estimates of effect sizes (e.g. Cohen's *d*, Pearson's *r*), indicating how they were calculated

Our web collection on statistics for biologists contains articles on many of the points above.

Software and code

Policy information about availability of computer code

Data collection	No software was used
Data analysis	Statistical analysis was performed using R and Python

For manuscripts utilizing custom algorithms or software that are central to the research but not yet described in published literature, software must be made available to editors and reviewers. We strongly encourage code deposition in a community repository (e.g. GitHub). See the Nature Portfolio guidelines for submitting code & software for further information.

Data

Policy information about availability of data

All manuscripts must include a data availability statement. This statement should provide the following information, where applicable:

- Accession codes, unique identifiers, or web links for publicly available datasets
- A description of any restrictions on data availability
- For clinical datasets or third party data, please ensure that the statement adheres to our policy

All analytical methods applied for the deep learning algorithm are included in this published article. De-identified data will be made available upon reasonable

request. Code is available at https://github.com/ecg-net/scd-oregon.

Human research participants

Policy information about studies involving human research participants and Sex and Gender in Research.

Reporting on sex and gender	We have provided sex distribution in the internal and external datasets in Table 1.
Population characteristics	We have provided demographic and clinical characteristics of our study subjects in Table 1.
Recruitment	We prospectively identified all potential sudden cardiac death (SCD) cases in two distinct USA communities (Portland Oregon metro area, Ventura County, California) in collaboration with each region's emergency medical services (EMS) system. Subsequently, in-house adjudication to confirm likely cardiac etiology was performed by trained physician-researchers using all available medical record data for each potential SCD case, EMS prehospital care reports, medical examiner's reports, and death certificates from Oregon and California state vital statistics records. All SCD cases with archived resting 12-lead ECGs available for analysis were included. These ECGs were recorded prior to and unrelated to the SCD event.<br><br>We recruited control subjects from the Portland Oregon metro area to represent corresponding patients from the general population. Control subjects were identified through multiple sources, including patients undergoing angiography, patients having their chest pain assessed by EMS, or patients visiting an outpatient cardiology clinic. We ascertained the control subjects so that the prevalence of CAD and MI was comparable to SCD cases. Control patients had no previous history of cardiac arrest or ventricular arrhythmias.<br><br>Pre-arrest clinical records and ECGs were available if the patient provided written consent or was deceased, in which case consent was waived.
Ethics oversight	Institutional review boards of Ventura County Medical Center, Oregon Health and Science University, Cedars-Sinai Health System, and all other relevant health systems and participating hospitals approved the study protocol.

Note that full information on the approval of the study protocol must also be provided in the manuscript.

Field-specific reporting

Please select the one below that is the best fit for your research. If you are not sure, read the appropriate sections before making your selection.

☒ Life sciences ☐ Behavioural & social sciences ☐ Ecological, evolutionary & environmental sciences

For a reference copy of the document with all sections, see nature.com/documents/nr-reporting-summary-flat.pdf

Life sciences study design

All studies must disclose on these points even when the disclosure is negative.

Sample size	We included all out-of-hospital SCD cases in the two communities (Portland Oregon metro area, Ventura County, California) with archived resting 12-lead ECGs available for analysis. Cases from Portland Oregon metro area (n=1,796) were used as internal dataset and were randomly divided into training, validation, and test cohorts while cases from Ventura County, California (n=714) were used to externally validate the model. A representative sample of ECGs from controls (n=1,325) were used to train and validate the model to identify SCD cases.
Data exclusions	In all study cohorts, ECGs with paced rhythm, atrial fibrillation, or atrial flutter were excluded a priori to create a DL model that could be applied to ECGs in sinus rhythm.
Replication	The model and results were externally validated using a geographically distinct community-based cohort of sudden cardiac death cases
Randomization	Cases in the internal cohort were randomly splitted into training, validation, and test cohorts
Blinding	Blinding was not relevant to train, test, and externally validate the deep learning model

Reporting for specific materials, systems and methods

We require information from authors about some types of materials, experimental systems and methods used in many studies. Here, indicate whether each material, system or method listed is relevant to your study. If you are not sure if a list item applies to your research, read the appropriate section before selecting a response.

Materials & experimental systems

n/a	Involved in the study
☒	☐ Antibodies
☒	☐ Eukaryotic cell lines
☒	☐ Palaeontology and archaeology
☒	☐ Animals and other organisms
☒	☐ Clinical data
☒	☐ Dual use research of concern

Methods

n/a	Involved in the study
☒	☐ ChIP-seq
☒	☐ Flow cytometry
☒	☐ MRI-based neuroimaging
